# Marked lateral deviation of the phrenic nerve due to variant origin and course of the thyrocervical trunk: a cadaveric study

**DOI:** 10.1007/s00276-015-1557-x

**Published:** 2015-10-05

**Authors:** Keiko Ogami, K. Saiki, K. Okamoto, T. Wakebe, Y. Manabe, T. Imamura, T. Tsurumoto

**Affiliations:** Department of Macroscopic Anatomy, Graduate School of Biomedical Sciences, Nagasaki University, 1-12-4 Sakamoto, Nagasaki, 852-8523 Japan; Department of Anesthesiology, Nagasaki University School of Medicine, Nagasaki, Japan; Department of Oral Anatomy and Dental Anthropology, Graduate School of Biomedical Sciences, Nagasaki University, Nagasaki, Japan

**Keywords:** Phrenic nerve, Diaphragm paralysis, Thyrocervical trunk, Subclavian artery, Variation

## Abstract

Phrenic nerve impairment can often lead to serious respiratory disorders under various pathological conditions. During routine dissection of an 88-year-old Japanese male cadaver, a victim of heart failure, we recognized an extremely rare variation of the right thyrocervical trunk arising from the subclavian artery laterally to the anterior scalene muscle. In addition to that, the ipsilateral phrenic nerve was drawn and displaced remarkably laterad by this vessel. We examined all of the branches arising from subclavian arteries, phrenic nerves and diaphragm muscles. The embryological background of this arterial variation is considered. The marked displacement with prolonged strain had a potential to cause phrenic nerve impairment with an atrophic change of the diaphragm muscle. Recently many image diagnostic technologies have been developed and are often used. However, it is still possible that rare variations like this case may be overlooked and can only be recognized by intimate regional examination while keeping these rare variations in mind.

## Introduction

The subclavian artery (SCA) is divided into three portions. The first portion is from the origin of the SCA to the internal side of the anterior scalene muscle (ASM), the second portion is behind the ASM, and the third portion is from the lateral margin of the ASM to the lateral border of the first rib.

The thyrocervical trunk (TCT) and the internal thoracic artery (ITA) are recognized as major branches of the SCA and they arise from the first portion. Generally, there are four branches of the TCT: the inferior thyroid artery (IFTA), the ascending cervical artery (ACA), the transverse cervical artery (TCA) and the suprascapular artery (SSA). These branches have numerous anatomical variations and originate from different portions of the TCT. The variation of the TCT is smaller than that of the other major branches of the SCA. The ITA arises from the lower side of the first portion of the SCA and is located on the opposite side of the TCT. The phrenic nerve (PN) passes near the TCT and the ITA. PN impairment can often lead to serious respiratory disorders under various pathological conditions.

In a particular dissection that we carried out, we noticed some unusual variations in the neck. The right TCT and ITA arose from the third portion of the SCA and his ipsilateral PN was drawn and displaced laterally to the TCT. We examined all the branches of the SCA, PNs and diaphragm muscles in both sides. We concluded these variations had embryological factors. We report these variations to bring attention to the possibility of other similar cases. In this way, we could understand the etiologic diagnosis and decide on courses of treatment for phrenic nerve paralysis as well.

## Case report

During a routine dissection of an 88-year-old formalin-preserved Japanese male cadaver, a victim of heart failure, the neck arteries were detected to have rare variations. The lengths and diameters of the arteries were measured via a Mitutoyo Caliper with accuracy value in 0.1 mm (Vernier Caliper; Mitutoyo, Kanagawa, Japan). This study was approved by The Ethics Committee of The Nagasaki University Graduate School of Biomedical Sciences.

There was no operative scar on the surface of the body. The branches of the aortic arch appeared normal trifurcation.

### The right side

**Fig. 1:**
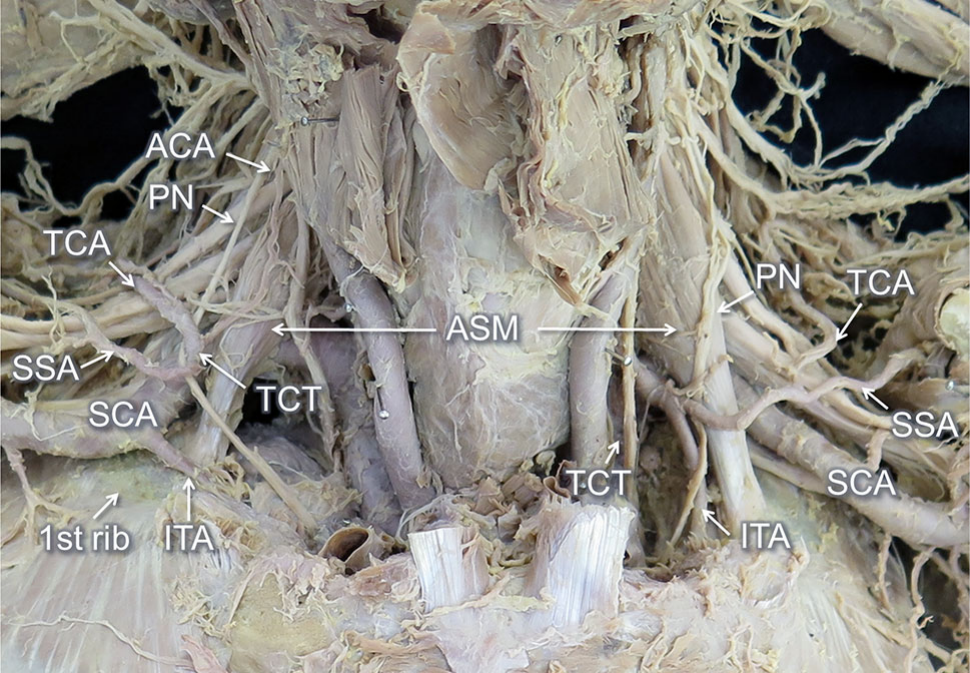
Frontal view of the neck. The veins, clavicles and surface muscles are already removed. The right thyrocervical trunk (TCT) and internal thoracic artery (ITA) originated from the third portion of the subclavian artery (SCA). The phrenic nerve (PN) is displaced laterad by the TCT. The right anterior scalene muscle (ASM) was smaller and the insertion was more laterally than the left one

**Fig. 2:**
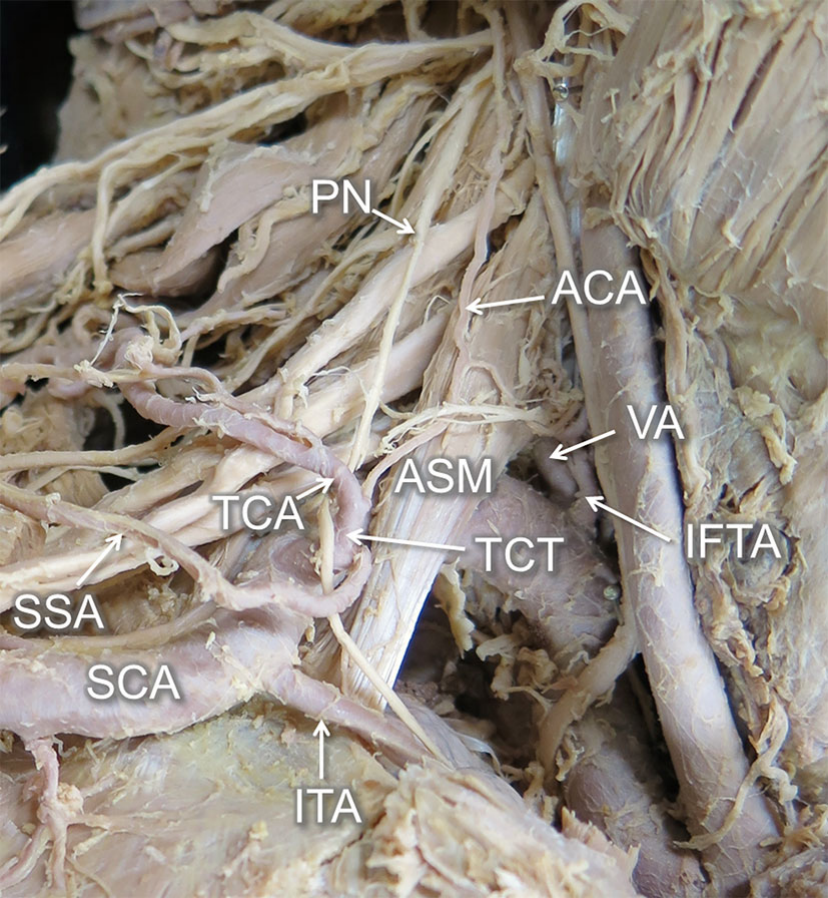
Enlarged view from the *right side*. The brachiocephalic artery divided into the right common carotid and SCA. In the first portion, the SCA ramified into two arteries—vertebral artery (VA) and inferior thyroid artery (IFTA). The root of TCT compressed the PN. It ramified trifurcately into the suprascapular artery (SSA), transverse cervical artery (TCA) and ascending cervical artery (ACA)

The right SCA originated at the point of 19.7 mm from the origin of the brachiocephalic artery, and at the first portion ramified the vertebral artery (37.2 mm from the origin of the SCA, with a 4.1 mm diameter) and the IFTA (39.2 mm from the origin of the SCA, with a 2.6 mm diameter) (Fig. 1). At the second portion ramified the costocervical artery (41.6 mm from the origin of the SCA, with a 3.0 mm diameter) and at the third portion ramified the TCT cranially (64.6 mm from the origin of the SCA, with a 4.8 mm diameter) and the ITA caudally (74.2 mm from the origin of the SCA, with a 3.2 mm diameter) (Figs. 1, 2). The right TCT ramified trifurcated for the suprascapular artery (4.7 mm from the origin of the TCT, with a 2.1 mm diameter), transverse cervical artery (6.1 mm from the origin of the TCT, with a 3.4 mm diameter) and ascending cervical artery (10.3 mm from the origin of the TCT, with a 1.4 mm diameter) (Fig. 2). The right PN arose independently from the fourth cervical nerve and descended passing the lateral side of the TCT toward the thorax. Therefore, the PN was pulled obviously laterally by the TCT. After passing the outside of the TCT, the nerve was bent at an angle of 60° and inserted to the thorax (Figs. 1, 2). The right ASM originated from the anterior tubercles of C5 and C6 and inserted to the first rib (61.9 mm laterally from the axis of the sternum). The diameter of the insertion was 15.6 mm and the thickness was 3.0 mm. The right lung collapsed with a pleural effusion because of some unknown reasons. Therefore, we could not observe the diaphragmatic elevation, which is one of the common symptoms of the diaphragm paralysis. The thickness of the diaphragm was 1.5 mm examined at 30 mm from the origin of the sternum: it was thinner than the left side (2.7 mm) measured at the symmetric point.

### The left side

The left SCA arose directly from the aortic arch. At the first portion ramified the vertebral artery (42.6 mm from the origin of the SCA, with a 11.2 mm diameter) and the TCT (47.5 mm from the origin of the SCA, with a 7.1 mm diameter) (Fig. 1). At the second portion ramified the costocervical artery (74.1 mm from the origin of the SCA). At the third portion, there were no arteries from the SCA. The left TCT ramified into three branches, the IFTA (4.4 mm from the origin of the TCT, with a 3.9 mm diameter), the ITA (5.2 mm from the origin of the TCT, with a 3.1 mm diameter), the common trunk of the transverse cervical artery and suprascapular artery (8.2 mm from the origin of the TCT, with a 2.2 mm diameter) and the ascending cervical artery (21.9 mm from the TCT, with a 1.6 mm diameter) (Fig. 1). The left PN arose from C3 to C5 and descended passing the lateral side of the TCT. The left ASM originated from the anterior tubercles of C3 to C6 and was inserted in the first rib (58.3 mm laterally from the axis of the sternum). The diameter of the insertion was 17.5 mm and the thickness was 5.6 mm. Many tumors and a plural effusion were observed in this side.

## Discussion

The TCT is one of the major branches of the first portion of the SCA. Generally, it has four branches: the IFTA, ACA, TCA, and SSA. These branches have numerous anatomical variations and originate from the different portions of the TCT. The variation of the origin of the TCT itself is smaller than that of the other branches of the SCA.

Concerning the origin of the TCT, Adachi did not find any cases in which the TCT arose from the third portion of the SCA in a total of 242 sides [1]. Daseler and Anson reported that in 770 sides there were no instances of TCT originating from the third portion [3]. Takafuji and Sato also reported that the TCT arose from the first portion of SCA in 94.4 % of 140 sides and from the second portion in 5.6 % with none originating from the third portion [10]. There have been only two reports of the TCT originating from the third portion of the SCA [7, 11]. Thomson described 1 case in 544 sides, but did not describe in detail [11]. Lischka et al. reported two cases in 166 sides, and in these instances the PNs descended to the thorax and went through the lateral side of the TCTs, similar to our case. One of these cases was in the right side, while it was a specific example where the artery was divided as the fifth branch of the aorta; this case belonged to the type CG of the Adachi–William–Nakagawa’s classification. The other case was the left side [7]. The phenomenon of the right TCT originating from the third portion of the ordinary SCA, like in our case, is extremely rare.

### Embryology

The transverse septum, which is a rudiment of the diaphragm, is located in the front of the third to fourth neck somite in the fourth week of intrauterine life. The transverse septum gradually shifts to the caudal side from the fifth week, and at this time it is accompanied by nerve fibers. As a result, the phrenic nerves originate from the anterior branch of C3–5 [5]. By the beginning of the eighth week, the dorsal part of the diaphragm is located at the level of the first lumbar [8].

In the seventh week of intrauterine life, the entire right SCA is formed from the right fourth aortic arch artery, the short portion of the right dorsal aorta and the right seventh intersegmental artery. On the other hand, the left SCA is formed from the left seventh intersegmental artery [9]. At around the same time, in the cervical region, the dorsal intersegmental arteries of either side are joined by a series of anastomoses to form the longitudinal channels. These longitudinal channels develop the branches of the SCA—the vertebral artery, ITA, supreme intercostal artery, deep cervical artery and ascending cervical artery [5]. The TCT is formed by the fusion of the IFTA, ascending cervical artery, transverse cervical artery and suprascapular artery [10].

From the fifth week, the myotomes rapidly start to develop. The scalene muscle is derived from the hypaxial portions of the cervical myotomes [2]. In the end of the seventh week of intrauterine life, they develop into the primordium of the scalene muscle. After a while, this muscle reaches the surface of the first rib, which develops an insertion point.

In our peculiar case, we speculate that the right anterior scalene muscle was inserted immediately lateral to the IFTA, and therefore the IFTA remained as an independent branch of the SCA, without making the normal TCT with the other arteries. The other three arteries were thought to fuse together as usual to form the TCT outside of the ASM accompanying the PN.

### Clinical implication

Kaufman et al. reported three patients with diaphragm paralysis caused by the TCA compression of the phrenic nerve. These arteries originated from the TCT in the first portion of the SCA. They named this condition as ‘Red Cross syndrome’. One of the three patients suffered from severe dyspnea when she turned her head to the affected side. Vascular decompression surgery on the patients had good outcomes [6].

In our case, unlike the Kaufman’s report, the root of TCT arising from the third portion of the SCA compressed the PN. We were not able to demonstrate the right PN and diaphragm paralysis. In addition to that, unfortunately, we could not reveal the symptoms during his lifetime. However, based on the change of posture and the movement of the neck and upper limb, the phrenic nerve might be pulled more by the ipsilateral TCT. This might have caused temporary dyspnea similar to Kaufman’s report.

Hamada et al. described that it is necessary to be aware of the supraclavian triangle blow Erb’s point during neck dissection procedures [4]. In the present case, these variations of the arteries and the PN originating and existing more laterally than usual could increase the risk of arterial and nerve puncture during internal jugular or subclavian venous catheter placement, neck surgery and brachial plexus block. The surgeons should check preoperative examinations including imaging tests, such as contrast-enhanced CT and MRI, in order not to damage these arteries and nerves. In addition, the use of an ultrasound-imaging device during such clinical procedures would improve the safety.

In conclusion, we encountered some rare variations, such as the right TCT and ITA originating from the third portion of the SCA and the ipsilateral phrenic nerve having been pulled and displaced laterally by the TCT, in an 88-year-old Japanese male cadaver. We studied these rare variations and considered the embryological and clinical analysis.

At an early stage of embryonic development, the branches of the SCA are formed from the longitudinal channels linking to the cervical intersegmental arteries. It was presumed that during this period, there was interference in the insertion of the ASM, which caused the TCT to displace remarkably laterad than usual. It is quite possible that this caused some episodes of dyspnea. This might have caused temporary dyspnea in this case. Recently many image diagnostic technologies, such as multiple directed computed tomography, have been developed and are often used. However, it is still possible that rare variations, like this case, may be overlooked and can only be recognized by intimate regional examination. These rare variations should be kept in mind due to this study.
